# Single-stage surgical repair in a complex case of aberrant right subclavian artery aneurysm and common carotid trunk

**DOI:** 10.1186/1749-8090-8-112

**Published:** 2013-04-25

**Authors:** Ioannis Kokotsakis, Leanne Harling, Vania Anagnostakou, Dimitris Tassopoulos, Christos Charitos, Hutan Ashrafian, Thanos Athanasiou

**Affiliations:** 1 Department of Cardiac Surgery, Evangelismos General Hospital Athens, Athens, Greece; 2 Department of Surgery and Cancer, Imperial College London, 10th floor QEQM Building St. Mary’s Hospital Praed Street, London, W2 1NY, UK

**Keywords:** Aberrant right subclavian artery aneurysm, Common carotid trunk, Open surgical repair

## Abstract

Aberrant right subclavian artery with coexisting common carotid trunk is an extremely rare congenital anomaly affecting <0.1% of the population. We report the case of a 77-year-old Caucasian man presenting with dysphagia and dyspnea secondary to an aberrant right subclavian artery aneurysm and describe our technique for open surgical repair.

## Background

Aberrant right subclavian artery (ARSA or arteria lusoria) is a congenital anomaly of the aortic arch affecting 0.4-2.3% of the population [1]. Common carotid trunk is present in 20-30% of these cases although is extremely rare amongst the general population (<0.1%) [2-4]. Aneurysms of the ARSA are often asymptomatic, however may present clinically with chest pain, dyspnea and/or dysphagia resulting from displacement of the trachea and oesophagus as the vessel courses through the posterior mediastinum.

In light of the high risk of rupture and subsequent mortality if left untreated, aneurysms of the ARSA (particularly in the presence of diverticulum of Kommerell) warrant prompt surgical or endovascular management [5,6]. We present a rare case of ARSA aneurysm with co-existing common carotid trunk and describe a technique for successful open surgical repair.

## Case report

**Figure 1:**
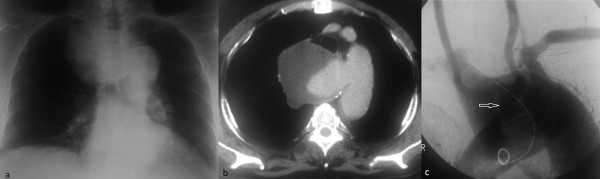
**Pre-operative imaging demonstrating aneurysmal aberrant right subclavian artery.** (**a**) Admission chest radiograph showing superior mediastinal mass (**b**) Computerised Tomographic images demonstrating CT a degenerative aneurysm of the ascending aorta and proximal left arch (**c**) Aneurysmal aberrant right subclavian artery.

A 77-year old Caucasian man presented with a 6-month history of intermittent dyspnea, dysphagia and upper chest pain. Physical examination revealed a soft diastolic aortic murmur and rales in the left lung base. His blood pressure was normal and symmetrical in the upper extremities. Blood tests were unremarkable with normal hepatic and renal function. Chest x-ray revealed a superior mediastinal mass, subsequently demonstrated on computerized tomography (CT) scanning to be a degenerative aneurysm of the ascending aorta (4.6 cm) and proximal left arch (4.0 cm), with an aneurysmal aberrant right subclavian artery (ARSA) (7.5 cm). The descending aorta was also moderately enlarged at 4.5 cm (Figure 1).

The aortic arch had a bovine configuration with common origin of the right and left common carotid artery. There was no innominate artery. The first cephalic artery was the common trunk (CT) of the right and left common carotid artery and the second the left subclavian artery (LSA). The ARSA arose from a Kommerell’s diverticulum just distal and inferiorly to the origin of LSA, which was aneurysmal and contained a large amount of calcified thrombus. It crossed the posterior mediastinum toward the right arm so that the trachea and esophagus were displaced anterolaterally (Figure 1c). Both vertebral arteries originated from subclavian arteries and had normal antegrade flow to the brain.

Preoperative coronary angiography revealed normal coronary arteries. Transthoracic echocardiography demonstrated moderate aortic regurgitation with a left ventricular ejection fraction of 50%. Carotid duplex scanning did not reveal any evidence of carotid artery stenosis.

### Surgical Procedure

**Figure 2:**
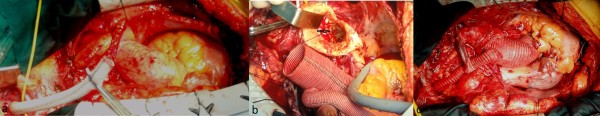
**Intra-operative images demonstrating our technique of open surgical repair.** Intra-operative images (**a**) Arterial cannulation introduced into the right common carotid artery via an interposed 8mm Dacron graft (**b**) Identification of Kommerell’s diverticulum and the origin of the right subclavian from within the aortic arch (**c**) The distal right subclavian artery (RSA) anastomosed to the side branch (10 mm) of the ascending aortic Dacron graft.

The heart and great vessels were approached by median sternotomy with the incision extended along the anterior border of the right sternocleidomastoid muscle to allow exposure of the right common carotid artery. After systemic heparinisation, CPB was instituted via a two-stage venous cannula in the right atrium and an arterial cannula introduced into the right common carotid artery through an interposed 8mm Dacron graft (Figure 2a). A retrograde cardioplegia catheter was placed in the coronary sinus, and LV decompression achieved via a right superior pulmonary vein vent. The patient was cooled to 25°C, during which time the ascending aorta, proximal aortic arch, CT and LSA were dissected and exposed without dividing the innominate vein.

Myocardial arrest was achieved by both retrograde and antegrade cold crystalloid cardioplegia (Custodiol). Aortic valve replacement was first performed using a 23 mm bioprosthetic valve (Trifecta, St.Jude Medical). A dacron vascular prosthesis with single side branch (30×10 mm) was then anastomosed to the sinotubular junction with external Teflon reinforcement.

At a temperature of 25°C, CPB flow was reduced to 1 L.min^-1^ maintaining an arterial pressure 50-60 mmHg. The CT of the right and left common carotid artery was clamped in order to obtain selective bilateral antegrade cerebral perfusion, with concomitant clamping of the LSA to avoid the steal phenomenon. Under systemic circulatory arrest, the residual ascending aorta and partial arch were resected sparing the origins of CT and LSA. Kommerell’s diverticulum and the origin of the ARSA aneurysm were identified and the diverticulum closed using an oval Dacron patch (Figure 2b). Distal anastomosis of the ascending aortic graft was then fashioned to the open hemi-arch with external Teflon reinforcement. Prior to completion of the distal anastomosis thorough de-airing was performed. Systemic circulation and was then resumed and rewarming commenced.

The right subclavian artery was transected proximal to the origin of the right vertebral artery. The aneurysm was partially resected, large amounts of thrombotic and atheromatous material evacuated, and the aneurysmal wall was closed in a continuous fashion. The distal right subclavian artery (RSA) was then routed below the innominate vein and anastomosed to the side branch (10 mm) of the ascending aortic Dacron graft (Figure 2c).

**Figure 3:**
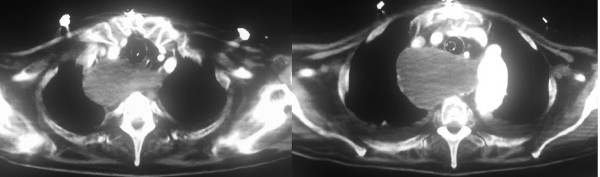
Post-operative CT scan demonstrating successful surgical reconstruction.

The patient was successfully weaned from CPB. The synthetic graft was excluded from the sternal wound by re-approximating the pericardium. The post-operative course was uneventful and follow-up CT two months after surgery demonstrated an excellent result (Figure 3).

## Discussion

ARSA is one of the most common congenital anomalies of the aortic arch, arising during development due to persistence of the right dorsal aorta (producing Kommerell’s diverticulum) and involution of the right 4^th^ aortic arch. Aneurysmal dilatation of the ARSA occurs in approximately 8% of cases, is usually degenerate in nature, and when symptomatic most commonly presents with chest pain, dysphagia and/or dyspnea.

In light of the high risk of rupture and subsequent mortality [5], early diagnosis and surgical or endovascular intervention for ARSA aneurysms is paramount [6]. Over recent years, advances in endovascular and hybrid therapy have led to less invasive treatment options in patients at high risk for open surgery [7,8]. Short-term outcomes from cervical anatomical debranching combined with aortic endograft placement are encouraging, with no significant complications, re-intervention or clinically significant endoleak reported at up to 2 year follow-up [8]. Furthermore, advances in branched endograft design may in future lead to a successful totally endovascular approach to complex arch aneurysms. However, despite encouraging early results, long-term outcomes for hybrid and endovascular procedures remain uncertain, and customized endovascular branched grafts may be costly and not widely available [8]. Furthermore, minimally invasive and hybrid approaches have at present been largely limited to the treatment of smaller, localized aneurysms. In our case the large aneurysm size, extensive involvement of the ARSA, ascending aorta and proximal left arch, and presence of substantial calcified thrombus precluded endovascular therapy.

Open surgical exposure may be via left thoracotomy, right thoracotomy or median sternotomy. Most commonly this involves a two-stage approach, firstly performing an extra-anatomical carotid-subclavian bypass followed by left thoracotomy and open thoracic aneurysm repair [9]. In this case, median sternotomy with an extended incision along the anterior border of sternocleidomastoid provided excellent exposure of both the arch and CCT and subclavian vessels permitting safe single-stage repair.

## Conclusions

Aneurysms of the aberrant right subclavian artery and Kommerell’s diverticulum are associated with a high risk of rupture and require definitive surgical management. Where aneurysmal dilatation involves both the ARSA, proximal and distal aortic arch, median sternotomy provides excellent exposure for safe open graft repair. The presence of common carotid trunk in up to 30% of these patients should be identified pre-operatively in order to provide optimal exposure and plan an appropriate perfusion strategy.

## Consent

Written informed consent was obtained from the patient for publication of this Case report and any accompanying images. A copy of the written consent is available for review by the Editor-in-Chief of this journal

## Abbreviations

ARSA: Aberrant right subclavian artery; CT: Computerised tomography; CCT: Common carotid trunk; CPB: Cardiopulmonary bypass

## Competing interests

There are no financial or non-financial competing interests.

## Authors’ contributions

IK, VA, DT and CC carried out the surgical procedure, participated in provision of clinical information and reviewed the manuscript. LH, HA and TA performed the literature review and drafted the manuscript. All authors read and approved the final manuscript.

## References

[B1] Yang C Shu C Li M Li Q Kopp R Aberrant subclavian artery pathologies and Kommerell's diverticulum: a review and analysis of published endovascular/hybrid treatment options Journal of endovascular therapy : an official journal of the International Society of Endovascular Specialists 2012 19 3 373 382 10.1583/11-3673MR.1 22788890

[B2] Murzi M Mariani M Tiwari KK Farneti P Berti S Karimov JH Glauber M Aberrant right subclavian artery aneurysm in coexistence with a common carotid trunk Ann Thorac Surg 2009 88 1 e8 10.1016/j.athoracsur.2009.04.110 19559178

[B3] Ozates M Nazaroglu H Uyar A MR angiography in diagnosis of aberrant right subclavian artery associated with common carotid trunk Eur Radiol 2000 10 9 1503 10.1007/s003300000335 10997446

[B4] Turkbey B Hazirolan T Canyigit M Peynircioglu B Cil BE Coexistence of aberrant right subclavian artery and common carotid trunk: Diagnosis with CT angiography European Journal of Radiology Extra 2007 62 2 63 64 10.1016/j.ejrex.2007.02.007

[B5] Fisher RG Whigham CJ Trinh C Diverticula of Kommerell and aberrant subclavian arteries complicated by aneurysms Cardiovasc Interv Radiol 2005 28 5 553 560 10.1007/s00270-003-0229-0 16091992

[B6] Stone WM Ricotta JJ 2nd Fowl RJ Garg N Bower TC Money SR Contemporary management of aberrant right subclavian arteries Ann Vasc Surg 2011 25 4 508 514 10.1016/j.avsg.2011.02.012 21549920

[B7] Chua CH Lin CH Hung CR Aneurysm of an aberrant right subclavian artery: treatment with thoracic aortic stent graft Ann Thorac Surg 2010 89 4 1289 1291 10.1016/j.athoracsur.2009.08.068 20338362

[B8] Knepper J Criado E Surgical treatment of Kommerell's diverticulum and other saccular arch aneurysms J Vasc Surg 2013 57 4 951 10.1016/j.jvs.2012.10.094 23332243

[B9] Kouchoukos NT Masetti P Aberrant subclavian artery and Kommerell aneurysm: surgical treatment with a standard approach J Thorac Cardiovasc Surg 2007 133 4 888 892 10.1016/j.jtcvs.2006.12.005 17382621

